# Barriers and facilitators to conducting economic evaluation studies of Gulf Cooperation Council (GCC) countries: a survey of researchers

**DOI:** 10.1186/s12961-021-00721-1

**Published:** 2021-05-01

**Authors:** Saja H. Almazrou, Shiekha S. Alaujan, Sinaa A. Al-Aqeel

**Affiliations:** Clinical Pharmacy Department, College of Pharmacy, King Saud University, Riyadh, Saudi Arabia

**Keywords:** Economic evaluation, Research, Barriers, Gulf Cooperation Council

## Abstract

**Background:**

The number of published economic evaluations of Gulf Cooperation Council (GCC) countries is notably scarce. Limited local evidence could have a major impact on the implementation of economic evaluation recommendations in the decision-making process in GCC countries. Little is known about the factors affecting researchers who seek to conduct economic evaluations. Therefore, we aimed to assess researcher barriers and facilitators in conducting such studies of GCC countries.

**Methods:**

A cross-sectional survey of health economic researchers working in GCC countries was conducted online between January and February 2020. The survey instrument collected researchers’ perceived barriers and facilitators and demographic information. For barriers, respondents rated their agreement on a 5-point Likert scale ranging from “strongly disagree” to “strongly agree”. For facilitators, respondents rated the importance of each facilitator on a six-point scale ranging from “extremely important” to “not very important”. Then, participants were asked to select the three most important barriers and facilitators from the lists. The data collected were examined using descriptive analysis.

**Results:**

Fifty-one researchers completed the survey (37% response rate). The majority of participants (more than 80%) agreed that lack of quality of effectiveness data and restricted access to unit cost data are the main barriers to conducting economic research. Availability of relevant local data was reported as an important facilitator, followed by collaboration between health economic researchers, policy-makers and other stakeholders.

**Conclusions:**

The results of this study provide an exploratory view of the issues faced by health economics researchers in GCC countries. Recommendations to GCC countries based on international experiences, such as to use real-world data in economic evaluation research, were provided.

## Background

The Gulf Cooperation Council (GCC) countries, which include the Kingdom of Saudi Arabia, the United Arab Emirates, Bahrain, Kuwait, Qatar and Oman, are considered by the World Bank to be high-income countries [1]. GCC countries share similar contextual and social factors, including language and religion [2]. These countries are members of the Gulf Health Council, which was established in 1976 to promote and improve the health sector for all member states by providing proactive initiatives and responding to regional and global health challenges [3]. The GCC healthcare sector is dependent on government funding, although mandatory insurance schemes are in various stages of implementation in the region.

Economic evaluation is a tool for comparing the costs and consequences of various interventions. The main goal is to provide information to decision-makers that can be used to improve the health of the population by ensuring the efficient use of available resources [4]. The ultimate aim of implementing an economic evaluation is to improve efficiency by optimizing resource allocation while providing the most effective interventions [5]. In addition, such an evaluation reduces the variation in health services provisioned across the country to ensure health equity [6]. Finally, economic evaluations are important in new technology price negotiations, developing clinical guidelines and public reimbursement lists [5]. Full economic evaluations include cost-effectiveness analyses, cost–utility analyses, cost–benefit analyses and cost minimization analyses, whereas partial evaluations include cost analyses and cost of illness.

According to a systematic review published in 2018, the first health economic study of a GCC country was published in 1991, and the number has continued to grow, reaching 49 studies in 2017 [7], with 40 studies (82%) classified as partial economic evaluations. The results from the abovementioned systematic review and other reviews [8] suggest that economic evaluations of the region are limited in quantity and quality [7–9]. This low publication rate is also observed in eastern Mediterranean countries [9] and low- and middle-income countries [10].

Limited local evidence could have a negative impact on the implementation of economic evaluations in the decision-making process in the region. Absence of relevant economic evaluations and difficulties translating economic evaluations into local decision contexts are commonly reported as barriers to using evidence from economic evaluations in the healthcare decision-making process [11–18]. Challenges in the transferability of economic evaluation result from differences between countries in clinical and cost data such as the incidence of a disease, clinical practice patterns, availability of health care resources, discount rates, and prices. Recommendations to assess the transferability of economic evaluations have been published [18, 19].

The pursuit of health sector efficiency and value for money are central dimensions of health care performance in GCC countries [20, 21]. For example, in Saudi Arabia, there has been an initiative to establish a health technology assessment (HTA) entity to achieve efficiency in resource allocation [22, 23]. HTA is a systematic tool that summarizes information regarding the medical, ethical and economic issues of a new health technology that requires research infrastructure and capacity.

In view of the limited number of published economic evaluations despite increasing interest in using economic evidence for resource allocation, a better understanding of issues that impede the conducting of economic evaluations will provide useful evidence that can be employed for the development of policies to promote the generation of health economics evidence. Researchers who are involved in conducting economic evaluations are in the best position to provide a perspective on the challenges they encounter that policy-makers may not be familiar with. This study aims to assess barriers and facilitators to conducting economic evaluations in GCC countries from the perspective of researchers.

## Methods

### Study design

A cross-sectional study using an online survey was carried out between January 1 and February 29, 2020.

### Study population

Researchers who published an economic evaluation of the GCC countries were the target population of this survey. We identified potential participants by referring to the reference lists of two systematic reviews of economic evaluations conducted on the GCC. We searched PubMed, Google, LinkedIn and ResearchGate to identify all authors’ contact information. We assumed that some GCC researchers may have conducted an economic evaluation but that their work was not identified by the previous two reviews; therefore, we identified researchers who contributed to the local health economics conference in the last 2 years. Finally, participants identified from reviews and conferences were asked to provide the contact details of other researchers in the field so that these individuals could also be invited to participate in the study.

### Instrument development and administration

The instrument consists of three sections. The first and second sections elicited participants’ perceived barriers and facilitators to conducting an economic evaluation, respectively. We are not aware of any published instruments on measuring barriers and facilitators to conducting economic evaluations. Therefore, we consulted studies assessing barriers and facilitators to the use of economic evaluations in decision-making [11, 16, 24–26], conducting randomized clinical trials in developing countries [27], and health research in general [28, 29] to compile two lists, one for barriers and one for facilitators. Three additional barriers and facilitators perceived by the authors, who all hold a PhD in health economics, were also listed. This process yielded a total of 21 barriers and 12 facilitators.

For barriers, respondents rated their agreement on a 5-point Likert scale ranging from “strongly disagree” to “strongly agree”. For facilitators, respondents rated the importance of each facilitator on a six-point scale ranging from “extremely important” to “not very important”. Then, participants were asked to select the three most important barriers and facilitators from the lists. This prioritization by respondents helps policy-makers focus on developing interventions to overcome the barriers and implement the facilitators with the greatest impact on the conducting of economic evaluations. Open-ended questions regarding any additional barriers and facilitators were available at the end of each section.

The last part of the questionnaire collected researchers' demographics, which included age, gender, qualifications, current number of economic evaluation publications and years of experience.

The face validity of the questionnaire was determined through two consecutive stages. The first stage was centred around gaining insights from two academics from the GCC region who had published several health service research papers. They were asked to review the content and the clarity of the language and length. A few comments about wording were received, and the survey was amended accordingly. The second stage involved review of the questionnaire by two experts in health economics: one based in Saudi Arabia and the second based in Kuwait. The reason for selecting two researchers from different countries was to ensure that the terminology used in the survey was applicable to all GCC countries. They were asked to comment on the readability of the questionnaire. In addition, the experts were asked to judge the content validity of the questionnaire by indicating if all relevant barriers and facilitators were included and suggesting new relevant items or eliminating irrelevant items. No comments or suggestions were made in the second stage.

The survey was developed using SurveyMonkey [30]. Eligible participants were sent an email that contained the information sheet and an invitation to participate in the study. The email contained a link to the electronic survey. When participants clicked on the link, their web browser opened the first page of the survey, which repeated the study information that was provided in the email. Participants had to check a box that stated, “I have read the information sheet and I agree to participate in this study survey, which will utilize the information for scientific research purpose” before proceeding to the next page. To increase the response rate, reminders were sent twice at one-week intervals. In addition, a charitable donation was granted for each completed survey. This study was approved by the King Saud University Medical City Institutional Review Board (KSUMC) (19/259/IRB).

### Data analysis

Descriptive statistics with percentages, mean and standard deviation, and median were used to demonstrate the most and least perceived barriers and facilitators provided by the researchers. Microsoft Excel [31] was used for the analysis.

## Results

### Participants

A total of 96 participants were identified by their publications, including the corresponding authors and all coauthors. Researchers identified 25 additional participants from local health economics conferences. The final number of researchers nominated by respondents was 22. No duplicates were identified among the nominated researchers.

Out of the 143 recruited respondents, 53 responded to the invitation (37%). The response rate classified by country is illustrated in Table 1. Among these respondents, 80% were males, and 60% were between 30 and 44 years old (Table 2).

**Table 1:** Response rate classified by country

Country	Number surveyed	Number responded	Response rate (%)
Saudi Arabia	92	40	43
Kuwait	6	2	33
UAE	17	4	24
Oman	16	5	31
Qatar	7	2	28
Bahrain	5	0	0
Total	143	53	37

**Table 2:** Participant characteristics (*n* = 51^a^)

Characteristics	*N* (%)		Characteristics	*N* (%)	
Age (years)			Number of published economic evaluation in the last 10 years		
18–29	9 (17.65)		0	7 (13.73)	
30–44	31 (60.78)		1–2	26 (50.98)	
45–59	8 (15.69)		3–6	15 (29.41)	
Above 60	3 (5.88)		More than 6	3 (5.88)	
Gender			Number of economic evaluations performed as part of job requirement in the last 10 years		
Male	42 (82.35)		0	9 (17.65)	
Female	9 (17.65)		1–2	13 (25.49)	
Nationality			3–6	13 (25.49)	
Australia	1 (1.96)		More than 6	16 (31.37)	
Canada	1 (1.96)		GCC country where you conducted most of your economic evaluations		
Egypt	1 (1.96)		Saudi Arabia	38 (74.51)	
India	1 (1.96)		Oman	5 (9.80)	
Jordan	1 (1.96)		United Arab Emirates	4 (7.84)	
Kuwait	2 (3.92)		Kuwait	2 (3.92)	
Oman	5 (9.8)		Qatar	2 (3.92)	
Saudi Arabia	34 (66.67)		Have you received any training courses in health economics?		
South Africa	1 (1.96)		Yes	40 (78.43)	
Sudan	2 (3.92)		No	11 (21.57)	
United States	2 (3.92)		Type of training		
Qualifications			Workshop	5 (12.20)	
Bachelor’s	8 (15.69)		Undergraduate course	1 (2.44)	
Master’s	14 (27.45)		Postgraduate course	3 (7.32)	
Doctorate	29 (56.86)		MSc\doctorate in health economics or pharmacoeconomics	32 (78.05)	
Years of experience			Affiliation		
0–5 years	17 (33.33)		Academic	21 (41.18)	
6–10 years	14 (27.45)		Hospital	10 (19.61)	
11–15 years	8 (15.69)		Public health institute	1 (1.96)	
16–20 years	4 (7.84)		Governmental research bodies	1 (1.96)	
More than 20 years	8 (15.69)		Ministry of health	8 (15.69)	
			Governmental bodies	4 (7.84)	
			Health industries and companies	6 (11.76)	

^a^Two participants did not complete the demographic section

### Barriers to conducting economic evaluations

The majority of participants (more than 80%) agreed that the lack of a health state preference to estimate quality-adjusted life-year (QALY) data is the main barrier to conducting economic evaluations, followed by the lack of, limited access to and poor quality of both cost and outcome data (Table 3).

**Table 3:** Barriers to conducting economic evaluations of GCC countries (*n* = 53)

Statements	Strongly agree % (*n*)	Agree % (*n*)	Neutral % (*n*)	Disagree % (*n*)	Strongly disagree % (*n*)	Summary	
						Mean (SD)	Median
Absence of relevant health state preference to estimate QALYs	52.8 (28)	37.7 (20)	5.6 (3)	1.8 (1)	1.8 (1)	4.38 (0.8)	5
Restricted access to unit cost datasets to value healthcare resource use such as costs of medications or diagnostics	41.5 (22)	37.7 (20)	15.0 (8)	5.6 (3)	0.0 (0)	4.15 (0.9)	4
Lack of local effectiveness data	45.2 (24)	35.8 (19)	5.6 (3)	9.4 (5)	3.7 (2)	4.09 (1.1)	4
Lack of quality effectiveness data including missing information, incomplete coding and misclassification of variables	39.6 (21)	43.4 (23)	5.6 (3)	9.4 (5)	1.8 (1)	4.09 (1.0)	4
Lack of an independent society for economic evaluation experts where they can meet and share their thoughts and overcome challenges	41.5 (22)	37.7 (20)	11.3 (6)	7.5 (4)	1.8 (1)	4.09 (1.0)	4
Lack of routinely collected national health statistics such as mortality classified by disease states and prevalence	39.6 (21)	41.5 (22)	5.6 (3)	13.2 (7)	0.0 (0)	4.08 (1.0)	4
Lack of contact and interaction among decision-makers, researchers and other stakeholders	30.1 (16)	47.1 (25)	15.0 (8)	5.6 (3)	1.8 (1)	3.98 (0.9)	4
Lack of skilled support personnel such as research assistants and researcher coordinators	39.6 (21)	32.0 (17)	13.2 (7)	15.0 (8)	0.00 (0)	3.96 (1.1)	4
Restricted access to routinely collected national health statistics such as mortality classified by disease states and prevalence	33.9 (18)	39.6 (21)	13.2 (7)	13.2 (7)	0.0 (0)	3.94 (1.0)	4
Fragmentation of the healthcare system; i.e., services are spread across many providers, making estimation of costs and outcomes difficult	39.6 (21)	30.1 (16)	16.9 (9)	11.3 (6)	1.8 (1)	3.94 (1.1)	4
Lack of financial support to conduct economic evaluation	33.9 (18)	33.9 (18)	15.0 (8)	15.0 (8)	1.8 (1)	3.83 (1.1)	4
Limited qualified human resources to conduct economic evaluation research	33.9 (18)	33.9 (18)	15.0 (8)	15.0 (8)	1.8 (1)	3.83 (1.1)	4
Lack of information on healthcare resources used by patients such as types and numbers of medications dispensed or diagnostic procedures performed	28.3 (15)	45.2 (24)	5.6 (3)	20.7 (11)	0.0 (0)	3.81 (1.1)	4
Lack of research infrastructure to support researchers (modelling and simulation software, skilled librarians, biostatisticians, research assistants)	26.4 (14)	43.4 (23)	16.9 (9)	11.3 (6)	1.8 (1)	3.81 (1.0)	4
Lack of support as decision-makers are unwilling to use economic evaluation findings in the decision-making	33.9 (18)	28.3 (15)	18.8 (10)	16.9 (9)	1.8 (1)	3.75 (1.2)	4
No methodological guidelines for conducting economic evaluation that is relevant to my country	22.6 (12)	39.6 (21)	20.7 (11)	7.5 (4)	9.4 (5)	3.58 (1.2)	4
Lack of support as decision-makers lack confidence in economic evaluation findings	16.9 (9)	39.6 (21)	20.7 (11)	15.0 (8)	7.5 (4)	3.43 (1.2)	4
Lack of researchers’ awareness of funding opportunities	9.4 (5)	49.0 (26)	18.8 (10)	18.8 (10)	3.7 (2)	3.42 (1.0)	4
Lack of researchers’ motivation to conduct economic evaluation research	16.9 (9)	37.7 (20)	11.3 (6)	16.9 (9)	16.9 (9)	3.21 (1.4)	4
Insufficient dedicated time for conducing economic evaluation research	11.3 (6)	30.1 (16)	30.1 (16)	24.5 (13)	3.7 (2)	3.21 (1.1)	3
Difficulties in obtaining ethical approval	5.6 (3)	18.8 (10)	32.0 (17)	26.4 (14)	16.9 (9)	2.7 (1.1)	3

Ten respondents added the following barriers in the open-ended question: lack of interest from decision-makers (*n* = 3), lack of collaboration with national authorities (*n* = 1), lack of local experts (*n* = 1), lack of publicly accessible data (*n* = 1), absence of a health economic society (*n* = 1) and lack of support to health economists (*n* = 1).

### Facilitators to conducting economic evaluations

Facilitators with the highest perceived importance scores (98%) were equally distributed among the following items: facilitating access to unit cost and effectiveness data; availability of a data warehouse containing details such as diagnoses and procedures performed in hospitals; and sufficient qualified human resources and expertise (Table 4).

**Table 4:** Facilitators to conducting economic evaluations of GCC countries (*n* = 51^a^)

Statements	Extremely important % (*n*)	Very important % (*n*)	Moderately important % (*n*)	Neutral % (*n*)	Slightly important %(*n*)	Not very important % (*n*)	Summary	
							Mean (SD)	Median
Accessibility to unit cost values for healthcare resources	64.7 (33)	25.5 (13)	7.8 (4)	0.0 (0)	0.0 (0)	2.0 (1)	5.49 (0.9)	6
Accessibility to routinely collected effectiveness datasets	51.0 (26)	41.2 (21)	5.9 (3)	2.0 (1)	0.0 (0)	0.0(0)	5.41 (0.7)	6
Availability of a data warehouse of all hospital admissions and outpatient appointments containing details, such as diagnosis and procedures performed	56.9 (29)	27.5 (14)	13.7(7)	0.0 (0)	2.0 (1)	0.0 (0)	5.37 (0.9)	6
Educate decision-makers about the value of economic evaluations in the decision-making process	60.8 (31)	17.7 (9)	13.7 (7)	7.8 (4)	0.0 (0)	0.0 (0)	5.31 (1.0)	6
Illustrating economic evaluation findings in a clear format to engage decision-makers	49.0 (25)	31.4 (16)	11.8(6)	7.8 (4)	0.0 (0)	0.0 (0)	5.22 (0.9)	5
Sufficient contact and interaction among health economic researchers, policy-makers and other stakeholders	43.1 (22)	39.2 (20)	9.8 (5)	5.9 (3)	2.0 (1)	0.0 (0)	5.16 (1.0)	5
Establishment of a centre for health economic research to inform policy and practice through conducting high-quality research	49.0 (25)	35.3 (18)	5.9 (3)	2.0 (1)	7.8 (4)	0.0 (0)	5.16 (1.2)	5
Establishment of an independent society for health economists	49.0 (25)	27.5 (14)	11.8 (6)	2.0 (1)	7.8 (4)	2.0 (1)	5.02 (1.3)	5
Sufficient qualified human resources and expertise	29.4 (15)	51.0 (26)	17.7 (9)	0.0 (0)	2.0 (1)	0.0 (0)	5.06 (0.8)	5
Sufficient support for researchers by providing professional development courses or workshops in health economics	27.5 (14)	41.2 (21)	27.5 (14)	2.0 (1)	2.0 (1)	0.0 (0)	4.9 (0.9)	5
Sufficient financial support to conduct economic evaluation research	29.4 (15)	37.3 (19)	21.6 (11)	7.8 (4)	3.9 (2)	0.0 (0)	4.8 (1.1)	5
Availability of methodological guidelines for conducting economic evaluation specific to my country	21.6 (11)	43.1 (22)	27.5 (14)	2.0 (1)	3.9 (2)	2.0 (1)	4.71 (1.1)	5

^a^Two participants did not complete the demographic section

This was followed by providing support to researchers in terms of expertise by conducting workshops and courses, enhancing decision-makers’ awareness of the value of economic evaluations and using simple methods to enhance their engagement with the results, scoring 96%, 92% and 92%, respectively.

Additional facilitators elicited from three participants through open-ended questions included collaboration between health economists in the GCC countries (*n* = 1), establishing a willingness-to-pay (WTP) threshold (*n* = 1) and introducing postgraduate programs in health economics.

### Top-rated barriers and facilitators

Participants were asked to select the most important barriers and facilitators from the provided list of 21 barriers and 12 facilitators. The top-rated barriers were the lack of local effectiveness data (*n* = 8), followed by restricted access to the unit cost dataset (*n* = 7) and a lack of quality effectiveness data (*n* = 7) (Table 5).

**Table 5:** Top-rated barriers and facilitators

	*N* (%)
Barriers (*n* = 53)	
Lack of local effectiveness data	8 (15.1)
Lack of quality effectiveness data	7 (13.2)
Restricted access to unit cost datasets to evaluate healthcare resources use	7 (13.2)
Absence of relevant health state preference to estimate QALYs	5 (9.4)
Lack of financial support to conduct economic evaluation research	4 (7.5)
Lack of routinely collected national health statistics	4 (7.5)
Other barriers	18 (34.0)
Facilitators (*n* = 51^a^)	
Availability of data warehouse of all hospital admissions and outpatient appointments	12 (23.5)
Establishment of a centre for health economic research	8 (15.69)
Accessibility to unit cost values for healthcare resources	6 (11.8)
Sufficient contact and interaction among health economic researchers, policymakers and other stakeholders	6 (11.8)
Other facilitators	19 (37.3)

^a^Two participants did not complete the ranking question

The most important facilitators were availability of a database of hospital admissions and appointments and accessibility of reported unit cost values (*n* = 12), followed by establishing a health economic research centre (*n* = 8) (Table 5).

## Discussion

In this cross-sectional study, barriers and facilitators to conducting economic evaluations of GCC countries were investigated. The findings suggest that the availability, quality and accessibility of local effectiveness and cost data are the main barriers.

One of the notable findings from this survey is the clear alignment between barriers and facilitators. The lack of and availability of data were rated as the top barrier and facilitator, respectively.

Few studies around the world have assessed challenges in using and conducting economic evaluations [16, 24, 25, 32]. Luz et al. [16] investigated the perceived challenges of conducting, using and reporting economic evaluations. Similar to our findings, they reported challenges with the availability and quality of the relevant data as the main technical issues. They also found that excluding economic evaluations from the decision-making process was one of the top-rated contextual barriers, i.e., the process and mechanism of translating evidence into policy, which mainly depends on policy-makers. This was also evident in our findings: 62% of respondents agreed that lack of support is a barrier, as decision-makers are unwilling to use economic evaluation findings in their decision-making process. This also echoes findings in other studies [7, 16, 25]. Challenges related to decision-makers’ support, confidence and understanding of the use of economic evaluations have also been addressed in other studies [24, 25, 32]. A study conducted by Roseboom et al. [25] found that decision-makers have some difficulties in interpreting economic evaluation outcomes, including QALYs, and in dealing with issues around the transferability of data across countries. Roseboom et al. [25] attributed this finding to the fact that only 20% of the respondents in their study had an economics background, whereas the remainder were from medical specialties.

One of the reported facilitators in our study was enhancing collaboration between researchers and policy-makers. In the Middle Eastern region, where GCC countries are located, El-Jardali et al. [33] reported that approximately 60% of researchers think that there is a lack of coordination between researchers and policy-makers. El-Jardali et al. [33] also reported limited coordination between different ministries and between government officials and healthcare providers, which ultimately hinders the health policy-making process. Therefore, decision-makers in GCC countries have a major role in bridging the gap between researchers and organizational bodies. This can be achieved by publicly inviting researchers from different sectors to participate in national projects either through health organization accounts in social networks or direct communication with universities and research centres. The establishment of a health economic research centre in GCC countries to foster a partnership between government, academia and the private sector, conduct policy-relevant economic evaluation research and contribute to training was suggested as a facilitator by the respondents.

Two thirds of the respondents highlighted the need for professional development courses or workshops in health economics, suggesting the need for short-course training programs as well as postgraduate training programs to increase the knowledge and skills required to conduct research in heath economics.

Future studies should investigate in depth the possible reasons for the barriers identified in our survey using qualitative research, including focus groups or interviews. The barriers and facilitators identified in our study should stimulate further research to design interventions and explain how these interventions are going to be implemented, i.e., implementation strategies. In addition, studies investigating the process of funding and reviewing economic evaluation reports are warranted.

## Strengths and limitations

This study has a number of strengths and limitations that are worth mentioning. To our knowledge, this survey is one of only a few studies assessing the barriers and facilitators to conducting health economics analyses. The method to identify the participants is a systematic, objective approach. Furthermore, asking participants to suggest potentially interested economic evaluation experts in their country to invite as fellow survey participants hopefully reduced the selection bias and increased the generalizability of the results.

The survey instrument was developed by the authors using published related evidence. A few new barriers and facilitators were added by the participants, which indicates the comprehensiveness of the listed barriers and facilitators. The survey was relatively short (the completion time was less than 10 minutes on average among our respondents).

Our survey included researchers from multiple countries. Therefore, using an online survey was deemed feasible and appropriate. Other known advantages of online surveys over other types are the flexibility and relatively low cost [34, 35].

Generally, using online survey methods has an inherent limitation, which is a low response rate compared with telephone or face-to-face surveys [36]. However, this response was expected in view of the low response rate reported in similar studies, which ranged between 19 and 35% [16, 33, 37]. In this study, we tried to enhance the response rate by sending multiple reminders at regular intervals, which significantly increased the response rate.

Another limitation of survey studies is that responses may represent either true beliefs and behaviours or perceptions about what respondents thought the questionnaire developers wanted to see, or a combination of both, which is also known as social desirability bias [38]. This could overestimate the importance of some factors over others. Finally, given that our survey was anonymous, we were not able to assess the difference between responders and nonresponders in terms of their demographic characteristics, introducing nonresponse bias into the study.

### Recommendations for GCC countries based on international experiences

In this study, there was agreement that the availability of clinical data was the main challenge for researchers in GCC countries. One of the effective data sources is randomized controlled trials (RCTs); however, the research capacity to conduct high-quality RCTs is still limited in the region and requires time and effort [39, 40]. Using real-world data (RWD) is a promising solution to conduct economic evaluations [41]. RWD are any type of data that are routinely collected in daily clinical practice; RWD offer several advantages, including a large population and long-term data, which is one of the main limitations of RCTs. A recent systematic review assessed the use of RWD in economic evaluations [42] and identified 93 studies that used RWD mainly from information systems such as administrative databases to identify direct medical costs and effectiveness outcomes, including survival rates. Despite the general limitations of RWD in terms of the presence of confounders and the amount of missing data, these data are a great alternative to RCTs. A successful experience of using RWD in economic evaluations has been studied in Germany [43]. In Germany, the most commonly used data source is insurance claims, and examples of effectiveness data include hospitalization, length of stay and mortality, whereas healthcare resource use and costs are mainly accessible for inpatient treatments. This indicates that a wide range of cost and clinical data can be gathered from routinely collected data with no extra cost and minimum effort.

The availability of a data warehouse of all hospital admissions and outpatient appointments as well as accessibility to unit cost and effectiveness datasets were among the facilitators suggested by the respondents. In the United Kingdom, where economic evaluations are better established, researchers have multiple sources to obtain health resource use and cost data [44]. The choice of sources depends on the type of analysis; for example, if the study is conducted in primary care, researchers are advised to access the Clinical Practice Research Datalink (CPRD) [45], whereas for studies conducted in secondary care, researchers should consult the Hospital Episode Statistics (HES) [46]. Another example is the National Cost Collection data, which cover aggregated costs and patient-level costs; this data source is also available for researchers in the United Kingdom. Access to these databases is regulated by the National Health Service (NHS digital), which is responsible for data protection and governance. Decision-makers should explore the advantages of establishing similar databases in GCC countries.

Healthcare resource use could also be collected using self-report methods such questionnaires and diaries. Another successful example of locating such instruments is the Database of Instruments for Resource-Use Measurement (DIRUM). DIRUM is also recommended by the International Society for Pharmacoeconomics and Outcomes Research to collect healthcare resource use data [47]. These instruments should be utilized by researchers in the GCC to collect resource use data from patients, caregivers or healthcare professionals.

Fragmentation of the healthcare system, i.e., the spreading out of services across many providers, which makes estimating costs and outcomes difficult, was identified as one of the barriers to conducting economic evaluations. In GCC countries, efforts to establish and develop electronic medical records (EMR) are still ongoing [48]. The fragmented nature of healthcare provisions in those countries means that the healthcare systems cannot capture the full patient journey, limiting the usefulness of this source to extract relevant data. However, there are some initiatives to consolidate and integrate EMR data distributed among different entities, i.e., private and government hospitals, into a single EMR [49–51]. To establish such a system, which is also known as health information exchange, different entities need to agree on the “minimum dataset”. This minimum dataset includes the specific domains, data elements, types and formats to be exchanged across the participating entities, patient information, provider information, services, medications and the classification codes, i.e., international classification of disease (ICD) codes, which serves as the “language” that allows the healthcare system to communicate. Agreeing on these standards and terminologies will ultimately enhance the quality of the retrieved data and reduce redundancies and duplications. All of this needs to be governed by a single entity, i.e., a ministry of health that dictates the rules and regulations for information sharing and privacy. After the medical information of each patient is integrated into a single medical record, the governing entity needs to establish data use agreement guidelines to ensure that the data are used legitimately for research purposes, minimizing the risk to patient privacy. This can include creating de-identified, multi-institution datasets after submitting the research proposal and obtaining institutional review board (IRB) approval.

## Conclusions

This study highlights that researchers in GCC countries strongly believe that a lack of local effectiveness data and accessibility to unit costs are the main barriers to conducting economic evaluations. Using real-world evidence is a promising solution that offers a timely and rich source of data in economic evaluations. One of the suggested facilitators was bridging the gap between researchers and decision-makers. This can be achieved by streamlining collaboration channels, such as by inviting researchers from different sectors to participate in national projects.

## Data Availability

All data generated or analysed during this study are included in this published article.

## Abbreviations

EMR: Electronic medical record
GCC: Gulf Cooperation Council
HTA: Health technology assessment
QALY: Quality-adjusted life-year
RWD: Real-world data
